# The safety of COVID-19 vaccines in patients with myasthenia gravis: A scoping review

**DOI:** 10.3389/fimmu.2022.1103020

**Published:** 2022-12-22

**Authors:** Siyang Peng, Yukun Tian, Linghao Meng, Ruiying Fang, Weiqian Chang, Yajing Yang, Shaohong Li, Qiqi Shen, Jinxia Ni, Wenzeng Zhu

**Affiliations:** ^1^ Department of Acupuncture, Guang’anmen Hospital, China Academy of Chinese Medical Sciences, Beijing, China; ^2^ Department of Acupuncture, Guang’anmen Hospital, Chinese Academy of Traditional Chinese Medicine Ji’nan Hospital (Ji’nan Hospital of Traditional Chinese Medicine), Shandong, China; ^3^ Department of Traditional Chinese Medicine, Yuyuantan Community Health Center, Beijing, China; ^4^ Treatment Center of Traditional Chinese Medicine, Beijing Bo’ai Hospital, China Rehabilitation Research Center, Beijing, China; ^5^ Treatment Center of Traditional Chinese Medicine, School of Rehabilitation, Capital Medical University, Beijing, China; ^6^ Department of Acupuncture, Dongzhimen Hospital of Beijing University of Traditional Chinese Medicine, Beijing, China

**Keywords:** myasthenia gravis, COVID-19, SARS-CoV-2, vaccines, safety

## Abstract

**Background:**

COVID-19 vaccines are required for individuals with myasthenia gravis (MG), as these patients are more likely to experience severe pneumonia, myasthenia crises, and higher mortality rate. However, direct data on the safety of COVID-19 vaccines in patients with MG are lacking, which results in hesitation in vaccination. This scoping was conducted to collect and summarize the existing evidence on this issue.

**Methods:**

PubMed, Cochrane Library, and Web of Science were searched for studies using inclusion and exclusion criteria. Article titles, authors, study designs, demographics of patients, vaccination information, adverse events (AEs), significant findings, and conclusions of included studies were recorded and summarized.

**Results:**

Twenty-nine studies conducted in 16 different countries in 2021 and 2022 were included. Study designs included case report, case series, cohort study, cross-sectional study, survey-based study, chart review, and systemic review. A total of 1347 patients were included. The vaccines used included BNT162b2, mRNA-1273, ChAdOx1 nCoV-19, inactivated vaccines, and recombinant subunit vaccines. Fifteen case studies included 48 patients reported that 23 experienced new-onset, and five patients experienced flare of symptoms. Eleven other types of studies included 1299 patients reported that nine patients experienced new-onset, and 60 participants experienced flare of symptoms. Common AEs included local pain, fatigue, asthenia, cephalalgia, fever, and myalgia. Most patients responded well to treatment without severe sequelae. Evidence gaps include limited strength of study designs, type and dose of vaccines varied, inconsistent window of risk and exacerbation criteria, limited number of participants, and lack of efficacy evaluation.

**Conclusion:**

COVID-19 vaccines may cause new-onset or worsening of MG in a small proportion of population. Large-scale, multicenter, prospective, and rigorous studies are required to verify their safety.

## 1 Introduction

It has been nearly three years since the outbreak of the coronavirus disease 2019 (COVID-19) pandemic caused by the severe acute respiratory virus coronavirus 2 (SARS-CoV-2) (1–4). The world is promoting COVID-19 vaccination in order to reduce infections and combat this pandemic (5). More than 100 vaccine candidates have reached clinical trials (6, 7). Currently, there are mainly four types of COVID-19 vaccines in use: nucleic acid mRNA-based vaccines, viral vector vaccines, inactivated virus vaccines, and subunit vaccines (8, 9).

The relationship between COVID-19 infection and myasthenia gravis (MG) exacerbation has been observed in numerous studies. In COVID-19-infected patients with MG, severe pneumonia and myasthenia crises are more frequent, and their mortality rate is considerably higher (10, 11). Thirty-six of 91 patients who had both MG and COVID-19 infection were reported to experience flare of symptoms and required rescue therapy (36/91, 39.56%), and 22 died due to COVID-19 (22/91, 24.18%) (10). This suggests that it is necessary to help patients with MG find ways to resist COVID-19 as the pandemic continues around the world. Currently, vaccination is the best way to achieve this goal. Vaccines approved for use have shown significant protective effect and safety in the general population with low incidence of adverse events (AEs) (12). Nevertheless, it is important to examine the safety and tolerability of these vaccines in specific medical conditions, such as MG, where there is still a concern that exacerbation of symptoms may occur after vaccination due to the immune response activation (13). Some case studies have reported a possible association between new-onset or worsening MG and vaccination. The proposed mechanisms for vaccine-provoked autoimmune diseases include molecular mimicry between the spike protein of SARS-CoV-2 and host self-antigens and bystander activation (14–16). Direct data on the safety and tolerability of COVID-19 vaccines in patients with MG are lacking, which results in hesitation in vaccination (17).

To collect and analyze the existing evidence on the safety of COVID-19 vaccines in patients with MG, understand the current research situation, and determine future research priorities and trends, we decided to conduct this scoping review. This scoping review was written in accordance with the PRISMA Extension for Scoping Reviews (PRISMA-ScR) (18). The protocol was registered and accessible to the public on the Open Science Framework (OSF) with registration DOI https://doi.org/10.17605/OSF.IO/C2ZE3.

## 2 Materials and methods

### 2.1 Determining the review questions

Before starting to conduct this review, we defined our broad exploratory research question as, “What has been studied about the safety of COVID-19 vaccines in patients with MG?” More specific research questions included: What types of studies have been conducted? What types of vaccines have been used? What are the results of safety evaluation? What are the directions and priorities for future studies?

### 2.2 Literature search

Three databases, including PubMed, Cochrane Library, and Web of Science (WOS), were systematically searched to identify all studies with MeSH terms including “myasthenia gravis,” “COVID-19,” “SARS-CoV-2,” “vaccines,” “vaccination,” and “COVID-19 vaccines.” The supplementary file presents the detailed search strategy. All clinical studies that investigated the safety profile of COVID-19 vaccines in patients with MG were included, including case reports, case series, randomized controlled trials, cohort studies, and reviews. No restrictions were placed on languages (from database inception to October 2, 2022). We excluded repeatedly published articles, conference abstracts, and dissertations.

### 2.3 Literature selection

All articles retrieved from the three databases were imported into EndNote software. After removing duplicate records, the titles and abstracts were independently reviewed by two investigators, and those that matched the inclusion criteria were kept for additional review. Subsequently, the full texts were read to assess for eligibility. When the two investigators disagreed with whether or not to include an article, they should have a discussion before final decision. If there was no consensus, a third researcher assisted in determining to include the article or not.

### 2.4 Data extraction

The extracted data included: article title, author, journal, country of origin, year of publication, objectives, population and medical history, study designs, type and dose of vaccines, outcomes, AEs, significant findings, and conclusions. An Excel table was designed in advance for the data extraction. To ensure accuracy, two investigators collected the data independently and checked them in real-time.

## 3 Results

### 3.1 Literature search results

**Figure 1:**
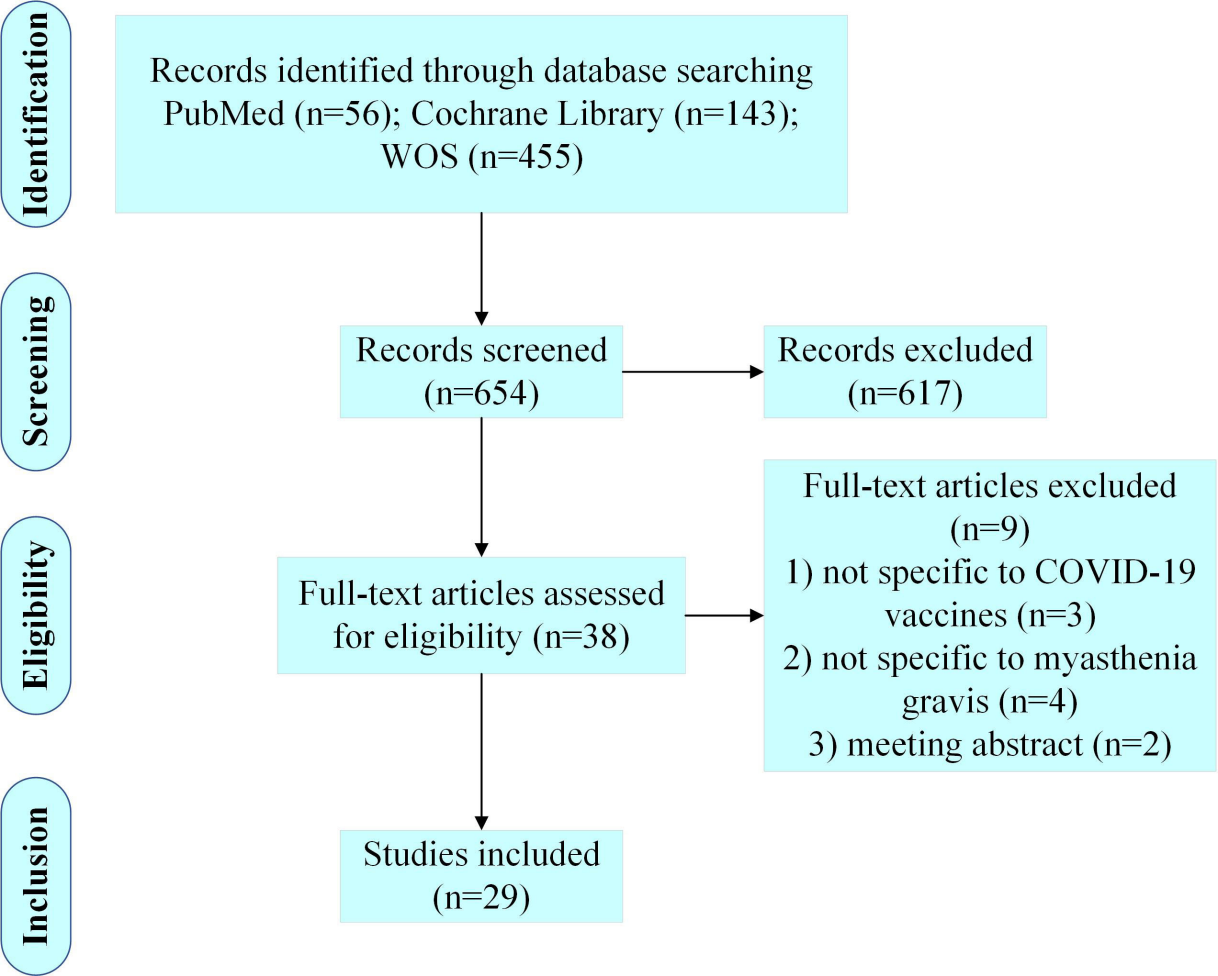
Diagram for scoping review literature identification.

A total of 654 articles were retrieved from the three databases, and 29 articles were included in this review after comprehensive literature screening (**Figure 1**).

### 3.2 General characteristics of the included studies

**Table 1** displays general features of the 29 studies, including publishing year, methodology, and country of origin. Three of the included studies were published in 2021, and 26 in 2022. In terms of study design, case studies (18/29, 62.07%) were the most frequent, including 14 case reports and four case series. Other types of study designs included three retrospective cohort studies, three prospective cohort studies, two retrospective survey-based studies, one systematic review, one retrospective chart review, and one cross-sectional study. The 29 studies were conducted in 16 different countries, with Italy (5/29, 17.24%), China (3/29, 10.34%), and the United States (3/29, 10.34%) conducting the most relevant studies.

**Table 1:** General characteristics of the included studies.

Variables	Categories	Number
Publication year	2022	26
	2021	3
Methodology	Case report	14
	Case series	4
	Retrospective cohort study	3
	Prospective cohort study	3
	Retrospective survey-base study	2
	Systematic review	1
	Retrospective chart review	1
	Cross-sectional study	1
Country	Italy	5
	China	3
	USA	3
	UK	2
	Spain	2
	Israel	2
	South Korea	2
	Japan	2
	Germany	1
	Thailand	1
	Australia	1
	KEN	1
	Canada	1
	Romania	1
	HRV	1
	Iran	1

### 3.3 Demographic characteristics of study participants

**Table 2** shows the detailed demographic and clinical features of the participants. In the 29 studies, 1347 participants in total were included, including 649 males (649/1347, 48.18%) and 698 females (698/1347, 51.82%). Four studies reported the mean onset age of the patients, and one study reported the median age at MG diagnosis. In the other 24 studies, the age of the patients ranged from 31 to 91 years. Only seven studies involving 769 patients reported the specific Myasthenia Gravis Foundation of America (MGFA) classification of participants. Among the 769 patients, 111 were asymptomatic, 152 were in grade I, 269 were in grade II, 174 were in grade III, 30 were in grade IV, and 33 were in grade V. Ten studies reported the detailed treatment for the patients before vaccination. The vaccines used in all included studies included BNT162b2 (Comirnaty, developed by BioNTech and Pfizer), mRNA-1273 (Spikevax, previously called Moderna, developed by Moderna Biotech), ChAdOx1 nCoV-19 (Vaxzevria, developed by AstraZeneca), inactivated vaccines, and recombinant subunit vaccines.

**Table 2:** Demographic and clinical characteristics of participants.

Author (year); country of origin	Study design	Sample size; (male/female)	Age (y)	MGFA classification	Flare (F)/New-onset(N)	Treatments before vaccination	Type of vaccine used
Dharmasaroja (2022); Thailand (19)	CR	N=1; (0/1)	31	IIa	1 F	Prednisolone 15 mg 1/2d, pyridostigmine 120 mg 3/d	BNT162b2
Fanella et al. (2022); Italy(20)	CS	N=3; (3/0)	91/80/55	NR	3 N	/	BNT162b2, mRNA-1273
Galassi et al. (2022); Italy(21)	CR	N=1; (1/0)	73	NR	1 N	/	ChAdOx1 nCoV-19
Huang et al. (2022); China(22)	CR	N=1; (1/0)	53	NR	1 N	/	ChAdOx1 nCoV-19
Kang et al. (2022); South Korea(23)	CR	N=1; (1/0)	35	NR	1 N	/	ChAdOx1 nCoV-19
Lee et al. (2022); South Korea(24)	CR	N=1; (1/0)	32	NR	1 N	/	BNT162b2
Sonigra et al. (2022); KEN(25)	CR	N=1; (1/0)	55	NR	1 F, MC	Prednisone 15 mg 1/2d, pyridostigmine 60 mg 1/d, AZA 150 mg 1/d	ChAdOx1 nCoV-19
Slavin et al. (2022); USA(26)	CR	N=1; (1/0)	60	NR	1 N	/	mRNA-1273
Abicic et al. (2022); HRV(27)	CR	N=1; (1/0)	65	NR	1 N	/	BNT162b2
Hoshina et al. (2022); Japan(28)	CR	N=1; (1/0)	30	NR	1 N	/	mRNA-1273
Maher et al. (2022); Australia (29)	CR	N=1; (1/0)	52	NR	1 N	/	ChAdOx1 nCoV-19
Poli et al. (2022); Germany (30)	CR	N=1; (1/0)	65	NR	1 N	/	BNT162b2
Ramdas et al. (2022); UK(31)	CS	N=7; (5/2)	Median age at diagnosis: 63	NR	7 N	/	BNT162b2, ChAdOx1 nCoV-19
Chavez et al. (2021); USA(32)	CR	N=1; (1/0)	82	NR	1 N	/	BNT162b2
Croitoru et al. (2022); Romania (33)	CR	N=1; (1/0)	78	IIb	1 N, MC	/	BNT162b2
Watad et al. (2021); UK(34)	CS	N=2; (2/0)	72/73	NR	2 N	/	BNT162b2
Tagliaferri et al. (2021); USA (35)	CR	N=1; (1/0)	77	NR	1 F, MC	Prednisone 7.5 mg 1/d, pyridostigmine 60 mg 6/d	mRNA-1273
Ruan et al. (2021); China(36)	CS	N=22; (16/6)	Mean onset age: 45.4	3 I, 1 IIb, 18 asymptomatic	2 F	12 AZA, 2 mycophenolate mofetil, 3 steroids combined with AZA	inactivated vaccines, recombinant subunit vaccines
Doron et al. (2022); Israel(37)	Cross-sectional study	N=160; (89/71)	57.2 ± 18	NR	9 F, 4 N	NR	BNT162b2
Farina et al. (2022); Italy(38)	Retrospective cohort study	N=104; (55/49)	Mean onset age: 57	21 I, 12 IIa, 19 IIb, 12 IIIa, 22 IIIb, 1 IVa, 8 IVb, 9 V	8F	69 corticosteroids, 21 AZA, 15 mycophenolate, 4 IVIG, 3 rituximab, 2 cyclosporine A	BNT162b2, mRNA-1273, ChAdOx1 nCoV-19
Ishizuchi et al. (2022); Japan(39)	Prospective cohort study	N=343; (119/224)	57	73 I, 128 II, 108 III, 10 IV, 24 V	3 F	240 prednisolone, 121 tacrolimus,26 cyclosporine, 93 IVIG, 102 methylprednisolone pulse therapy, 50 PE, 6 anti-complement	BNT162b2, mRNA-1273
Urra Pincheira et al. (2022); Canada (11)	Retrospective chart review	N=200; (103/97)	64.3 ± 13.9	NR	/	73 only pyridostigmine, 56 IVIG/PE, 91 one immunosuppressant agent, 30 two immunosuppressant agents, 7 three immunosuppressant agents	BNT162b2, mRNA-1273, ChAdOx1 nCoV-19
Sansone and Bonifati (2022); Italy(16)	Retrospective cohort study	N=80; (41/39)	60.4	NR	4 F	NR	BNT162b2, Mrna-1273, ChAdOx1 nCoV-19
Reyes-Leiva et al. (2022); Spain (40)	Prospective cohort study	N=100; (45/55)	Mean onset age: 55.85	52 asymptomatic, 13 I, 33 IIa, 2 IIIa	1 F	9 cholinesterase inhibitor monotherapy, 67 prednisone, 14 AZA, 21 mycophenolate mofetil, 6 cyclosporine, 7 tacrolimus, 9 rituximab, 3 IVIG, 1 PE	mRNA-1273
Lupica et al. (2022); Italy(41)	Retrospective cohort study	N= 53; (29/24)	63	NR	14 F	NR	BNT162b2, mRNA-1273, ChAdOx1 nCoV-19
Gamez et al. (2022); Spain(42)	Prospective cohort study	N=91; (36/55)	Mean onset age: 44.41 ± 20.97	13 I, 38 II, 29 III, 11 IV	2 F	42 pyridostigmine, 28 prednisone, 5 AZA, 5 mycophenolate, 58 tacrolimus, 9 IVIG	mRNA-1273, BNT162b2
Li et al. (2022); China(43)	Retrospective survey-base study	N=107; (54/53)	45.68 ± 1.49	41 asymptomatic, 29 I, 30 IIa, 6 IIb, 1 IIIa	11 F	NR	homologous inactivated vaccines, homologous recombinant subunit vaccines
Lotan et al. (2022); Israel(44)	Retrospective survey-base study	N=56; (35/21)	53	NR	8 F	28 corticosteroids 28, 4 AZA, 3 mycophenolate mofetil, 2 methotrexate, 9 rituximab, 6 IVIG, 2 PE, 1 eculizumab	BNT162b2
Mirmosayyeb et al.(2022); Iran (45)	Systematic review	N=5; (4/1)	66.6	NR	5 N	/	BNT162b2, ChAdOx1 nCoV-19

MGFA, Myasthenia Gravis Foundation of America; CR, case report; NR, not reported; MC, myasthenia crisis; AZA, azathioprine; IVIG, intravenous immunoglobulin; PE, plasma exchange.

### 3.4 Summary of case studies

**Table 3** presents detailed information of the included 18 case studies (19–36) involving 48 patients. Twenty-eight of the 48 participants experienced the onset or flare of MG, and three of them experienced myasthenia crisis. Twenty-three participants had new occurrences of MG: 12 (12/23, 52.17%) after receiving BNT162b2, six (6/23, 26.09%) after ChAdOx1 nCoV-19, four (4/23, 17.39%) after mRNA-1273, and one (1/23, 4.35%) after two doses of ChAdOx1 nCoV-19 and one booster dose of BNT162b2. Five patients had flares of MG symptoms after vaccination: one (1/5, 20.00%) after mRNA-1273, one (1/5, 20.00%) after BNT162b2, one (1/5, 20.00%) after ChAdOx1 nCoV-19, one (1/5, 20.00%) after the inactivated vaccine, and one (1/5, 20.00%) after the recombinant subunit vaccine. Four patients reported explicit onset or flare of symptoms after the first dose, nine after the second, and four after the third dose. The exact time of onset or flare ranged from the evening of vaccination to four weeks after each dose. Common MG symptoms after vaccination included dysarthria, perioral numbness, fatigability, dysphagia, dysphonia, diplopia, and ptosis. Fifteen studies reported that the patients responded well to treatment, and two studies did not report the results of the patients after the intervention. One study reported the death of a patient due to myocardial infarction, organ failure, and myasthenia crisis after receiving one dose of ChAdOx1 nCoV-19 (25). The results of case studies showed that there is a possible association between COVID-19 vaccination and worsening or new-onset MG. However, further studies and data are needed to support this finding.

**Table 3:** Detailed information of case studies.

Author; study design	Population (male/female, age[y]); medical history	Type of vaccine	Time of onset	Flare (F)/New-onset(N)	Symptoms and positive examination	Intervention and outcome	Significant findings
Dharmasaroja; CR (19)	N=1 (0/1, 31);/	BNT162b2	12 d after 2nd dose, 4 d after thymectomy surgery	F	Weakness, diplopia, ptosis, dysarthria	Prednisolone 20 mg 1/d, pyridostigmine 120 mg 4/d; recovered	It is uncertain whether thymectomy performed soon after vaccination contributed to MG aggravation, further investigation is needed.
Fanella et al.; CS (20)	N=1 (1/0, 91); chronic IHD, CKD	BNT162b2	10 d after 2nd dose	N	Fatigability, head drop, ptosis, diplopia, limitation in the upward gaze; RNS: facial nerve, 58%↓, AChR-Ab: 10.05 nmol/L	Hospitalization, pyridostigmine 30 mg 3/d; 1 m later, symptoms unchanged	Whether the vaccine is related to the development of MG is not clear.
	N=1 (1/0, 80); hypertension, T2DM, hypercholesterolemia	mRNA-1273	6 d after 2nd dose	N	Ptosis, diplopia, dysphagia, head drop; ice pack test (+), RNS: facial nerve 49%↓, AChR-Ab: 9.00 nmol/L	Hospitalization, pyridostigmine 30 mg 3/d, prednisone 25 mg 1/d, PE, AZA 25 mg to 150 mg 1/d; residual mild ptosis in right eye	
	N=1 (1/0, 55); hypertension, CTTH	mRNA-1273	3 d after 1st dose, worsened after 2^nd^ dose	N	Weakness, fatigability, diplopia, neck pain, orthopnea; RNS: facial and spinal accessory nerve 51%↓, AChR-Ab: 6.10 nmol/L	Hospitalization, pyridostigmine 60 mg 4/d, IVIG; mild upper limb fatigability, no bulbar and ocular involvement	
Galassi et al.; CR (21)	N=1 (1/0, 73); hypertension, MI, psoriasis	ChAdOx1 nCoV-19	8 d after vaccination	N	Myalgias, fever, ptosis; RNS: innasalis muscle 14.7%↓, AChR-Ab: 1.90 nmol/L	Pyridostigmine bromide 240 mg 1/d; symptoms improved	/
Huang et al.; CR (22)	N=1 (1/0, 53); hypertension	ChAdOx1 nCoV-19	1 d after 1st dose	N	Diplopia, diarrhea, ptosis, neck weakness, tingling numbness; Cogan lid twitch (+), AChR-Ab: 16.965 nmol/L, RNS: accessory nerve↓	Pyridostigmine 120 mg 3/d, prednisolone 15 mg 1/d; symptoms improved	The case highlights a possible association of COVID-19 vaccination and MG.
Kang et al.; CR (23)	N=1 (1/0, 35);/	ChAdOx1 nCoV-19	1 w after vaccination	N	Vertical binocular diplopia; ice packing test (+), AChR-Ab: 1.60 nmol/L	/	COVID-19 vaccination may cause MG with ocular symptoms, mechanism requires investigation.
Lee et al.; CR (24)	N=1 (0/1, 33);/	BNT162b2	the evening of 2nd dose	N	Ptosis, diplopia, dysarthria, dysphagia, weakness; myalgia neostigmine test (+), RNS: orbicularis↓, thymic hyperplasia	Hospitalization, pyridostigmine 360 mg 1/d; ptosis, dysarthria, and muscle strength improved	This case of new-onset MG is related to the vaccination and vaccination could trigger early-onset MG symptoms.
Sonigra et al.; CR (25)	N=1 (1/0, 55); T2DM, hypertension	ChAdOx1 nCoV-19	2 w after 1st dose	F, MC	Breathlessness, chest pain, sweating, cold clammy extremities, dysphagia, dysarthria; ECG: sinus tachycardia and N-STEMI, echocardiogram: hypokinetic anterior wall, interventricular septum, and apex	Admitted to ICU, enoxaparin sodium, prednisone, pyridostigmine, IVIG, percutaneous tracheostomy; MC could not be reversed, succumbed from MI and organ failures	There is a possible association between vaccination and MC in MG patients, enhanced monitoring and intervene at the earliest time are needed.
Slavin et al.; CR (26)	N=1 (1/0, 60); hypertension, hyperlipidemia, SVT, T2DM with peripheral neuropathy	mRNA-1273	6 d after 3rd dose	N	Dysarthria, diplopia, dysphagia, ptosis, dyspnea, fatigue, weight loss; ANA (+), edrophonium challenge (+), RNS: facial nerve 10%↓	Hospitalization, nasogastric tube, pyridostigmine; NR	Due to the proximity of time and no other known factors, we must be suspicious of an association between vaccination and onset of MG.
Abicic et al.; CR (27)	N=1 (1/0, 65);/	2 doses of ChAdOx1 nCoV-19, 1 booster dose of BNT162b2	3 w after booster dose	N	Vertical diplopia; AChR-Ab: >800 nmol/L	Intravenous MP 1000 mg 1/d for 5 d, pyridostigmine 180 to 300 mg 1/d, prednisone 10 to 20 mg 1/d; symptoms improved	Diplopia following vaccination should raise suspicion of new-onset OMG.
Hoshina et al.; CR (28)	N=1 (1/0, 30);/	mRNA-1273	2 d after 1st dose	N	Blurred vision with horizontally displaced images, arm weakness; ice-pack test (+), neostigmine test (+), AChR-Ab: 0.3 nmol/L	Pyridostigmine 30 mg 3/d, prednisone 10 mg 1/d; symptoms improved but fluctuated	mRNA-1273 vaccination may exacerbate subclinical cases of MG.
Maher et al.; CR (29)	N=1 (1/0, 52); hypertension, T2DM, refractive surgery for bilateral myopia	ChAdOx1 nCoV-19	1 d after 2nd dose	N	Diplopia, ptosis, extraocular movement limitations, muscle fatigability; ice pack test (+), SFEMG: abnormal jitter (orbicularis oculi)	Pyridostigmine 60 mg 5/d, prednisone 50 mg 1/d; ocular weakness improved	This is a case of OMG likely induced by the vaccine, the benefits of vaccination to protect against potential deadly consequences outweigh risk in possible onset or exacerbation.
Poli et al.; CR (30)	N=1 (1/0, 65);/	BNT162b2	3 d after 3rd dose	N	Fluctuating diplopia, ptosis of the right eye; AChR-Ab: 2.10 nmol/L	Pyridostigmine 90 mg 2/d; NR	The rare occurrence and favorable outcomes of vaccine-triggered MG do not detract from the public health imperative to vaccinate.
Ramdas et al.; CS (31)	N=7 (5/2, mean age at diagnosis: 63); 1 patient had a family history of AID, 1 patient had thyroid disease	5 received BNT162b2, 2 received ChAdOx1 nCoV-19	4 w after vaccination	N	5 patients diagnosed with GMG and 2 with OMG; 6 patients with AChR-Ab (+), 2 with RNS (+), 2 with SFEMG (+)	4 required nasogastric feeds, 1 required intensive care support for pneumonia and respiratory failure, 2 required IVIG and 1 required PE; all patients responded well to treatment	There is a possible association between vaccination and new-onset MG in a small proportion of susceptible individuals.
Chavez et al.; CR (32)	N=1 (1/0, 82); laryngeal cancer post hemi-laryngectomy, Barrett’s esophagus, 3a CKD	BNT162b2	2 d after 2nd dose	N	Dysarthria, perioral numbness, difficulty in chewing or spitting, ptosis, weakness, aspiration pneumonia; AChR-Ab: 11.4 nmol/L; RNS: decrement (+)	Hospitalization, pyridostigmine, speech therapy, IVIG, steroids, ventilator, PEG; recovered	Diagnosis of vaccine associated new-onset MG requires awareness of less common symptoms including dysarthria and dysphagia, and attention to timing of vaccination.
Croitoru et al.; CR (33)	N=1 (1/0, 78);/	BNT162b2	16 d after 3rd dose, 9 d after COVID-19 diagnosis	N, MC	Diplopia, asymmetrical bilateral ptosis, dysphonia, dysphagia, muscle weakness; ice-pack test (+), neostigmine test (+), RNS: right nasalis 17.3-20.8%↓, AChR-Ab: 19.20 nmol/L	Pyridostigmine 240 mg 1/d, prednisone 20 mg 1/d, 2 m later MC happened, admitted to ICU, intubated, IVIG, methylprednisolone 64 mg and pyridostigmine 240 mg per day; after 15 d, extubated, discharged	The first case of MG clinically manifested after COVID-19 infection and third dose SARS-CoV-2 vaccination. Even though at the moment no direct causality relationship can be established between these 2 disorders.
Watad et al.; CS (34)	N=1 (1/0, 72); recurrent pericarditis	BNT162b2	1 d after 2nd dose	N	RNS: facial and shoulder muscles 28-46%↓	Hospitalization, PE, prednisone 60 mg; quick symptom improvement	MG flares or onset associated with vaccination appear rare, most are moderate in severity and responsive to therapy.
	N=1 (1/0, 73);/	BNT162b2	7 d after 2nd dose	N	EMG: markedly pathologic jitter	Hospitalization, PE, intubation	
Tagliaferri et al., CR (35)	N=1 (1/0, 77);/	mRNA-1273	1 w after 2nd dose	F, MC	Dysphagia, arthralgia, fevers, chills, fatigue, lethargy	Pyridostigmine 60 mg 6/d, prednisone 7.5 mg 1/d, IVIG; another MC, intubation, prednisone increased to 40 mg, another two IVIG; extubated	Given the history of the vaccine coinciding with the onset of dysphagia, it is likely that the exacerbation was attributed to the vaccination.
Ruan et al.; CS (36)	N=22 (16/6, mean onset age 45.4); 7 underwent thymectomy, 4 confirmed thymoma	21 received inactivated vaccines, 1 received recombinant subunit vaccine	within 4 w after vaccination	F	19 patients with AChR-Ab (+)	2 slight symptoms worsening, increased the dose of pyridostigmine; resolved quickly	Inactivated vaccines might be safe in patients with MGFA classification I to II, supporting the recommendation to promote vaccination.

CR, case report; CKD, chronic kidney disease; AChR-Ab, anti-acetylcholine receptor antibody; RNS, repetitive nerve stimulation; IVIG, intravenous immunoglobulin; PEG, percutaneous endoscopic gastrostomy; CS, case series; PE, plasma exchange; EMG, electromyography; MC, myasthenia gravis; IHD, ischemic heart disease; T2DM, type 2 diabetes mellitus; AZA, azathioprine; CTTH, chronic tension-type headache; MI, myocardial infarction; ECG, electrocardiography; N-STEMI, ST-elevation myocardial infarction; AID, autoimmune disease; GMG, generalized myasthenia gravis; OMG, ocular myasthenia gravis; SVT, supraventricular tachycardia; MP, methylprednisolone; MGFA, Myasthenia Gravis Foundation of America.

### 3.5 Summary of other studies

**Table 4** presents detailed information of the included 11 articles other than case studies, involving 1299 patients. The criteria for defining exacerbation or onset of MG varied among the studies. Common criteria included changes in scales, such as myasthenia gravis activities of daily living scale (MG-ADL) or quantitative myasthenia gravis scale (QMG), symptom changes and duration, and treatment escalation. The window of risk ranged from seven days to six weeks after each vaccine dose. Ten studies reported that 60 participants (60/1299, 4.62%) experienced exacerbations, and nine had new-onset MG (9/1299, 0.69%). One retrospective chart review reported no symptom flares in 200 patients with MG after vaccination (11). Common AEs reported in these studies included local pain, fatigue, asthenia, cephalalgia, fever, and myalgia. The following are brief introductions of the included studies:

**Table 4:** Detailed information of other studies.

Author; study design	Sample size (male/female, mean age[y])	Type of vaccine; window period after each dose	Outcomes	Significant findings	Conclusion
Doron et al.; cross-sectional study (37)	N=160 (89/71, 57.2 ± 18)	BNT162b2; 6 w	Patients-reported exacerbation, exacerbation was confirmed by an increase ≥ 2 points in MG-ADL; AEs	13 worsening and confirmed in 8 patients (4 new-onset); the rate of exacerbations does not exceed the rate in previous years without exposure to the vaccine; mild post-vaccination exacerbations and AEs; no hospitalization	The vaccination did not raise the risk for exacerbation or new-onset of MG.
Farina et al.; retrospective cohort study (38)	N=104 (55/49, onset age: 57)	BNT162b2, mRNA-1273, ChAdOx1 nCoV-19; 4 w	Worsening was defined as the reoccurrence of symptoms or signs of muscle weakness lasting ≥ 24 h; MGFA grading; PIS classification	Minor AEs; 8 mild worsening, 6 regressed spontaneously, 1 increased steroid, 1 reached CSR after increasing steroids, IVIG and rituximab; no differences in the MGFA/PIS before and after vaccination	The data support the safety and tolerability of mRNA COVID-19 vaccines.
Ishizuchi et al.; prospective cohort study (39)	N=294 (119/224, 57)	BNT162b2, mRNA-1273; the day of 1st dose to 28 d after 2nd dose	MGC, MG-ADL, MG-QOL-15; flare was defined as: ≥ 7 d of disease, relapse of ≥ 3 points on the MGC, and treatment escalation	3 female patients experienced flare and responded well to IVIG and/or IVMP without sequelae; no fatal AEs	The relative risk of COVID-19 vaccines is exceedingly low.
Urra Pincheira et al.; retrospective chart review (11)	N=200 (103/97, 64.3 ± 13.9)	BNT162b2 (73.5%), mRNA-1273 (12.0%), ChAdOx1 nCoV-19 (6%), mixed vaccines (8.5%); 2 w	Primary: vMGII, SSQ, PASS; secondary: AEs	vMGII, SSQ, and PASS remained stable after the 1st and 2nd vaccinations; mild AEs	COVID-19 vaccinations were not associated with worsening severity of MG. The prevalence of AEs was the same as in the general population.
Sansone and Bonifati; retrospective cohort study (16)	N=80 (41/39, 60.4)	BNT162b2 (85.00%), mRNA-1273 (6.25%), ChAdOx1 nCoV-19 (3.75%), unidentified (5.00%); the day of 1st dose to 6 w after 2nd dose	Mild exacerbations: requiring steroid/immunosuppressant introduction/dose increase; severe exacerbations: PE/IVIG	4 patients developed exacerbation after the 2nd dose of BNT162b2. They fully recovered after steroid dose increase.	COVID-19 vaccination might rarely lead to disease worsening. The benefits outweigh by far the potential risks.
Reyes-Leiva et al.; prospective, cohort study (40)	N=100 (45/55, onset age: 55.85)	mRNA-1273; 1 w	changes in symptoms were considered relevant with an increase ≥ 3 points in MG-ADL, AEs, seroconversion and T-cell immune response rates	Exacerbation confirmed in 1 patient; mild AEs occurred in 14 patients after 1st dose and 21 after 2nd dose; 87 patients developed a humoral response and 72 showed a T-cell response.	The mRNA-1273 vaccination is safe and shows no negative impact on the disease course.
Lupica et al.; retrospective cohort study (41)	N=53 (29/24, 63)	BNT162b2 (90.56%), mRNA-1273 (7.55%), ChAdOx1 nCoV-19 (1.89%); 3 w	AEs, worsening was defined as ≥ 2 points increase in MG-ADL	14 patients experienced worsening while no significant difference in the MG-ADL before and after vaccination (p = 0.20); mild AEs with complete resolutions within 1 w	Vaccines against SARS-CoV-2 showed good short-term safety in MG patients.
Gamez et al.; prospective cohort study (42)	N=91 (36/55, onset age: 44.41 ± 20.97)	mRNA-1273, BNT162b2; 7 d	QMGS, MG-ADL, PIS	58.2% of the patients developed one or more transient AEs; 2 developed mild deteriorations	The mRNA vaccines do not induce exacerbations and have high levels of safety in stable patients with MG.
Li et al.; retrospective survey-based study (43)	N=107 (54/53, 45.68 ± 1.49)	105 with inactivated vaccines, 2 received recombinant subunit vaccines; 28 d	Worsening was defined as the occurrence of additional symptoms or the aggravation of existing symptoms in at least 1 of the 6 muscle groups (extraocular, facial, bulbar, cervical, limb and respiratory muscles)	11 patients reported 12 episodes of worsening after vaccination, including 6 after the 1st dose and 6 after the 2nd dose. A significantly higher incidence of worsening in patients with interval since last aggravation ≤ 6 m (P = 0.046).	COVID-19 vaccines (the majority were inactivated vaccines) were safe in milder MG patients. Worsening was more frequently seen in patients who were presumed as unstable.
Lotan et al.; retrospective survey-based study (44)	N=56 (35/21; 53)	BNT162b2; NR	AEs, neurological symptoms	55 patients received the vaccine, 19 reported immediate AEs. 8 reported new-onset or worsening of symptoms, including muscle weakness, dysphagia, dyspnea, and diplopia. 3 increased the dose of corticosteroids. 1 reported the persistency of symptoms.	The safety of the BNT162b2 vaccine in MG is similar to that reported in general population. The rate of AEs may be lower in MG patients treated with immunotherapies. The rate of worsening neurological symptoms is low.
Mirmosayyeb et al.; systematic review (45)	N=5 (4/1; 66.6)	BNT162b2 (80%), ChAdOx1 nCoV-19 (20%)	AEs, new-onset of MG	The main treatments of patients were pyridostigmine and prednisone. 1 patient was intubated while 4 recovered.	The onset of MG has been associated with the vaccine in a small number of cases. It cannot be conceded that MG is necessarily a complication of the vaccines, and more data and cases are needed.

PIS, postintervention status; CSR, complete symptom resolution; MGC, myasthenia gravis composite; MG-ADL, myasthenia gravis activities of daily living; MG-QOL-15, revised 15-item myasthenia gravis quality of life scale; PSL, prednisolone; IVMP, methylprednisolone pulse therapy; vMGII, virtual myasthenia gravis impairment index; SSQ, single simple question; PASS, patient acceptable symptom state; NR, not reported; AE, adverse event.

Doron et al. (37) conducted a cross-sectional study including 160 participants in an Israeli hospital to examine the safety of BNT162b2 vaccines. A questionnaire was sent to document whether they had an exacerbation of symptoms (confirmed by a minimum two-point increase on the MG-ADL), AEs, duration of exacerbations, and treatment change within 6 weeks after each dose. One hundred and fifty participants received the vaccines and 13 patients (13/150, 8.70%), including nine previously diagnosed MG and four new-onset MG, reported worsening myasthenia symptoms, and exacerbation was confirmed in eight patients (8/150, 5.30%). The flares lasted for less than three weeks in six patients, over three months in six patients, and one had an undeterminable duration. No hospitalization was necessary for any of these exacerbations, and three patients with previously diagnosed MG had increased prednisone dosages. In the 2021 vaccination period, seven patients reported an exacerbation, compared to six patients in the control period in 2020 (*P* = 0.880). The data showed that the exposure to vaccines did not lead to a significant increase in the rate of MG exacerbations. According to the electronic medical records of this hospital, there were 14 cases of newly diagnosed MG that visited the hospital in 2021, 19 in 2020, 27 in 2019, and 15 in 2018. The notion that the incidence of MG and vaccination are positively correlated is not supported by these data. Only mild AEs, such as flu-like symptoms, local pain, or isolated fatigue with a short duration, were reported. The findings support repeated vaccination in patients with MG.

Farina et al. (38) conducted a retrospective cohort study to investigate the safety of COVID-19 vaccines in 104 patients with MG from two centers in Italy. Ninety-eight (98/104, 94.20%) participants received at least two doses of vaccines four weeks before the survey, and 63 of them (63/98, 64.20%) received the booster dose. The vaccines with frequent use included BNT162b2, mRNA-1273, and ChAdOx1 nCoV-19. Worsening was defined as the reoccurrence of symptoms or signs of muscle weakness lasting for at least 24 hours. Minor AEs such as local pain and fever were reported. Mild MG worsening after vaccination was observed in eight cases (8/104, 7.70%), mostly after BNT162b2 (7/8, 87.50%). The frequency of worsening among anti-muscle-specific tyrosine kinase antibody-positive MG (MuSK-MG) cases (3/9, 33.30%) was significantly higher than that among anti-acetylcholine receptor antibody-positive MG (AChR-MG) cases (4/83, 4.80%) and seronegative cases (1/12, 8.30%). Six of the eight patients experienced spontaneous regression of symptoms (6/8, 75.00%). After receiving the second dose of mRNA-1275, one seronegative patient who had diplopia, limb weakness, and bulbar deficiency required an increase in steroids dose. One MuSK-MG patient experienced complete resolution of symptoms after addition of intravenous immunoglobulin (IVIG) and rituximab. Generally, the findings favored of the safety and tolerability of the COVID-19 vaccines in patients with MG.

Ishizuchi et al. (39) conducted a prospective cohort study involving 343 patients with MG in Japan. A total of 294 patients (294/343, 85.70%) received COVID-19 vaccines, BNT162b2 in 254, and mRNA-1273 in 40. No fatal AEs were reported. Three female patients with early onset MG (3/294, 1.02%), two who received BNT162b2 and one who received mRNA-1273, experienced flare, two with the generalized form, and one with the ocular form. The timing of onset varied from two days after the first dose to 14 days after the second dose. The patients reported a mean increased scores of the myasthenia gravis composite (MGC) from three to 12, MG-ADL from two to nine, and revised 15-item myasthenia gravis quality of life scale (MG-QOL-15) from four to 15, indicating that worsening of symptoms severely impaired quality of life. The patients responded well to IVIG and/or methylprednisolone pulse therapy (IVMP) without further sequelae. The data indicated that the COVID-19 vaccination had an exceedingly low risk. However, postponing vaccination may be safer for patients with severe symptoms, such as bulbar involvement or myasthenia crisis. It is important to keep a close eye on symptom changes, especially in the first week following vaccination.

Urra Pincheira et al. (11) conducted a retrospective chart review in Canada involving 200 patients who received two vaccine doses. In total, 147 patients received BNT162b2 (147/200, 73.50%), 24 received mRNA-1273 (24/200, 12.00%), and 12 received ChAdOx1 nCoV-19 (12/200, 6.00%). Seventeen patients received mixed vaccines (17/200, 8.50%). There were no significant differences in the virtual myasthenia gravis impairment index (vMGII), patient acceptable symptom state (PASS), and single simple question (SSQ) before and after vaccination. Nearly 90.00% of the patients were PASS yes at the end of the study, with a mean SSQ value of 82.00% and mean vMGII of seven, indicating a well-controlled population. None of the patients required hospitalization. About 60.00% of individuals after the first dose and 56.00% after the second dose, respectively, reported AEs, such as injection site pain and fatigue.

Sansone and Bonifati (16) conducted a retrospective cohort study in Italy involving 80 patients who received two doses of COVID-19 vaccines. 68 patients received BNT162b2 (68/80, 85.00%), five received mRNA-1273 (5/80, 6.25%), three received ChAdOx1 nCoV-19 (3/80, 3.75%), and four received an unidentified type of vaccine (4/80, 5.00%). Sixty-nine patients (69/80, 86.25%) did not show any exacerbation within the window period of risk. It was difficult to identify an exacerbation in seven patients (7/80, 8.75%) during the window period because of plasma exchange (PE) or IVIG treatment on a regular basis. Four patients (4/80, 5.00%) had MG exacerbation after receiving the second dose of BNT162b2, two patients required steroid/immunosuppressant introduction or dose increase with rapid control of the symptoms, and two required PE/IVIG. These data suggest that COVID-19 vaccines rarely result in worsening MG. As some studies have shown that the combination of COVID-19 and MG might exacerbate either of the two, the researchers concluded that the benefits of vaccination outweigh the potential risks.

Reyes-Leiva et al. (40) performed a prospective cohort study that included 100 participants in Spain to determine the safety profile of mRNA-1273 vaccines in patients with MG. All participants received two mRNA-1273 vaccine doses. The researchers collected the MG-ADL scores seven days before and seven days after each dose. Symptom changes were considered to be relevant to the vaccination if the MG-ADL increased three points or more. Eight (8/100, 8.00%) and ten (10/100, 10.00%) patients reported a significant increase in MG-ADL after the first and second doses, respectively. In these 18 patients, after the first dose, the mean MG-ADL increase was 3.25 points, and after the second dose, it was 3.90 points. This worsening was self-limiting in 17 participants, and they were not considered to have an exacerbation. The remaining patient with generalized myasthenia gravis (GMG) experienced a four-month exacerbation after the second dose. The MG-ADL score increased by three points, and the patient required treatment modification. Fourteen (14/100, 14.00%) participants reported mild AEs after the first dose, and 21 (21/100, 21.00%) after the second dose. No association was found between the rates of AEs and immunosuppressive treatment. The results of this study verified the safety of vaccination.

Lupica et al. (41) performed a retrospective cohort study involving 53 patients with MG who received two COVID-19 vaccine doses in Italy. Exacerbation was confirmed if the MG-ADL score increased two points or more before and after vaccination. Forty-eight patients received BNT162b2 (48/53, 90.57%), four received mRNA-1273 (4/53, 7.54%), and one received ChAdOx1 nCoV-19 (1/53, 1.89%). The MG-ADL score increased in 14 patients (14/53, 28.30%) after vaccination, which was more common in females compared than in males (*P* = 0.048). However, there was no substantial difference in the MG-ADL score prior to and following vaccination in all patients. (*P* = 0.20). Two patients (2/53, 3.77%), seven patients (7/53, 13.21%), and 15 patients (15/53, 28.31%) reported AEs after the first, second, or both doses, respectively. Common AEs included local pain, asthenia, cephalalgia, fever, and myalgia. The results of this study support the use of COVID-19 vaccines in patients with MG.

Gamez et al. (42) conducted a prospective cohort study involving 91 patients in Spain to investigate the safety of mRNA vaccines in patients with well-controlled MG. Thirty-eight patients (38/91, 41.80%) developed at least one transient adverse event. No significant association was found in the rates of AEs, or steroid use or dose change. Two patients (2/91, 2.20%) experienced changes in functional status, and none of the patients experienced myasthenia crisis. The first patient experienced diplopia (QMG from nine to 11 and MG-ADL from three to five) lasting four days, and no treatment modification was required. The second patient experienced lower limb fatigue (QMG from four to seven and MG-ADL from one to six) lasting two weeks, and the prednisone dose was increased. The data from this study support the safety of COVID-19 vaccines in patients with well-controlled MG.

Li et al. (43) performed a retrospective survey-based study involving 107 patients to investigate the safety of inactivated COVID-19 vaccines in China. Worsening was defined as the appearance of new symptoms or aggravation of pre-existing symptoms in at least one of the extraocular, facial, cervical, bulbar, limb, or respiratory muscles within 28 days after each dose. One hundred and seven patients received at least one dose (105 received inactivated vaccines and two received homologous recombinant subunit vaccines). Eleven participants (11/107, 10.28%), including six after the first dose and six after the second dose, experienced 12 bouts of deterioration in total after vaccination. Only one female patient required an increase in prednisone dose, as well as the addition of tacrolimus, and she recovered two months later. In patients who were deemed unstable (with an interval since last aggravation less than six months), the exacerbation rate was noticeably higher (*P* = 0.046). The only independent factor correlated to exacerbation, according to logistic regression, was the interval since last worsening less than six months (*P* = 0.01). The data from this study showed that inactivated vaccines are safe for milder patients. Patients who were thought to be potentially unstable experienced worsening more frequently. After vaccination, patients presumed to be stable also showed mild worsening.

Lotan et al. (44) conducted a survey-based study in Israel documenting immediate AEs and the new-onset or worsening of neurological symptoms in patients with MG to investigate the safety and tolerability of the BNT162b2 vaccines. Fifty-five patients received vaccines, 51 received two doses, and four received one dose. Nineteen patients (19/55, 34.55%) reported immediate AEs, such as pain at the injection site, fatigue, headache, and dizziness following vaccination, four after the first dose, six after the second dose, and nine after both doses. AEs were more commonly seen in patients aged < 55 years. Eight patients (8/55, 14.55%) experienced occurrence or exacerbation of neurological symptoms, such as muscle weakness, dysphagia, dyspnea, dysarthria, ptosis, and diplopia, four after the first dose, two after the second dose, and two after both doses. Three patients experienced symptom onset or worsening within a few hours after the vaccine, four after one to four days, and one after one week. One of the eight patients had to increase the dose of corticosteroids. Six patients reported symptom relief within a month, while two experienced persistent symptoms for more than a month. The researchers concluded that the safety profile of BNT162b2 vaccine in patients with MG is comparable to that in the general population. Patients receiving immunotherapy may experience fewer AEs.

Mirmosayyeb et al. (45) conducted a case report-based systematic review to investigate whether MG is a complication of COVID-19 vaccines. Four studies that reported five new-onset MG cases were included. Their study found that the onset of MG was associated with vaccination in a few cases. However, due to the lack of sufficient data, more studies and cases are required before reaching a conclusion.

## 4 Discussion

### 4.1 Summary of findings

Our scoping review identified 29 studies to examine the existing evidence on the safety of COVID-19 vaccines in patients with MG. The article title, author, journal, country of origin, year of publication, purpose of the study, population and medical history, methodology, type and dose of vaccines, outcomes, AEs, significant findings, and conclusions were recorded. The 29 studies were conducted in 16 different countries and included 1347 participants. Case studies included 48 participants and 28 of them experienced new-onset or worsening of MG after vaccination, suggesting that there may be an association between the onset or flare of MG and COVID-19 vaccines. Ten observational studies and one systemic review reported 60 exacerbations (60/1299, 4.62%) and nine new-onsets (9/1299, 0.69%) of MG. Common AEs included local pain, fatigue, asthenia, cephalalgia, fever, and myalgia. The data suggest that only a minority of patients who received the vaccines reported worsening as well as mild AEs, supporting the safety of COVID-19 vaccines in the MG population. However, most studies still called for more prospective, multicenter, large-scale, and rigorously conducted studies to provide strong evidence and verify their safety.

### 4.2 Gaps in the evidence and research priorities

#### 4.2.1 Limited strength of study designs

There were too many case studies and retrospective studies in all of the included articles. There could be a significant publication bias as negative findings are usually not published in an article. Thus, case studies can provide relatively limited evidence to support the safety of COVID-19 vaccines in patients with MG. However, the accumulation of cases demonstrates that potential risks exist between vaccination and MG onset or flare. Case studies can also provide meaningful lessons and references to clinicians in understanding the clinical features of MG after vaccination, window of risk, treatment, and prognosis. Recall errors are common in retrospective studies and may cause bias in the results of the studies, leading to limited strength of evidence. Therefore, more prospective and rigorously-designed studies are required in the future.

#### 4.2.2 Type and dose of vaccines varied

Currently, most studies have evaluated the safety of mRNA vaccines because of their broad availability. The safety of different types of vaccines deserves further detailed research because people in different countries and regions can only access certain types of vaccines. For example, inactivated vaccines are mainly used in China, but their safety in patients with MG is under-evaluated. The participants in the 29 studies received different doses of vaccines, ranging from one to three doses. At present, many countries are promoting intensive vaccination, and the potential risks of different doses or boost vaccination need to be observed in depth and comprehensively evaluated in the future.

#### 4.2.3 Inconsistent window of risk and exacerbation criteria

The window of risk observed after vaccination in different studies was different. Some studies chose six weeks as the risk period because this period was considered a vaccine-related effect in studies in the context of Guillain-Barre syndrome, and influenza vaccination (46), and MG exacerbation after influenza vaccination (47). The criteria for MG exacerbation after vaccination also differed. Some studies required clinicians to make objective judgments according to changes in scales, while some studies had relatively subjective criteria for aggravation. The standardized and unified window of risk and aggravation criteria are the basis for accurately evaluating the relationship between vaccination and new-onset or flare of MG. If the observed window of risk is exceeded or the specified standard is not met, caution should be exercised before concluding that there is a relationship between MG flares or onset and vaccination.

#### 4.2.4 Limited number of participants

The number of patients included in all studies was small, which limits the reliability of the conclusions. To obtain highly reliable conclusions, observational studies require a large sample size. Due to the heterogeneity of MG, whether the safety of vaccines for different MG populations is consistent deserves further study. If patients with different severities, antibody types, immunosuppressive treatment schemes, and different levels of stability have different risks or AEs after vaccination, more large-sample, in-depth research and analysis should be conducted in these fields.

#### 4.2.5 Lack of efficacy evaluation

Only a few studies have investigated the protective effect of COVID-19 vaccines in patients with MG when evaluating the safety. As the pandemic seems to have a severe impact on patients with MG, in addition to the risks and AEs of vaccination, attention should be paid to evaluating the effectiveness of vaccines against SARS-CoV-2 infection. The benefits and risks should be compared objectively to provide more reasonable instructions to patients with MG in clinical practice.

### 4.3 Strength and limitations

This scoping review comprehensively searched articles about the safety of COVID-19 vaccines in patients with MG in three databases using standard literature retrieval methods, and summarized the research results on this topic. This study had several limitations. First, since the main purpose of this review was to collect and organize all the evidence, the quality of the included studies was not evaluated. Second, no specific conclusions could be drawn to answer more detailed questions because no analysis was conducted on the data of the included studies. Third, as the main purpose of this scoping review was to provide a comprehensive summary of evidence relating to safety, we refrained from assessing the effectiveness of vaccines against SARS-CoV-2. Finally, the researchers conducting this review were all clinicians. If basic researchers were involved, they might have a more in-depth view of this review.

## 5 Conclusion

This scoping review provides an overview of the evidence that is currently available about the safety of COVID-19 vaccines in patients with MG. Case studies have demonstrated that COVID-19 vaccines may cause new-onset or worsening of MG in a small proportion of the population. Some retrospective and prospective observational studies have reported only a few AEs or worsening of MG, supporting the safety of COVID-19 vaccines in patients with MG. Large-scale, multicenter, prospective, and rigorous studies are needed to verify their safety.

## Author contributions

WZ, JN, and LM screened the literature. WC, YT, and QS extracted data of all the studies. SP wrote the draft of the review. SL, YY, and RF revised the manuscript. All authors contributed to the article and approved the submitted version.
