# Supplementary material for: The safety of COVID-19 vaccines in patients with myasthenia gravis: A scoping review
Source: Front Immunol. 2022 Dec 22;13:1103020. doi: 10.3389/fimmu.2022.1103020 (PMC9812949; doi:10.3389/fimmu.2022.1103020)
Supplement: Supplementary file 1 [file DataSheet_1.docx]

Search strategy for Pubmed

| No. | Search (untagged = all searchable fields which includes titles, abstracts, and index terms) | Records Retrieved |
| --- | --- | --- |
| 1 | Myasthenia Gravis[MeSH Terms] | 16602 |
| 2 | (((((Myasthenia Gravis) OR (MG)) OR (Ocular Myasthenia Gravis)) OR (Generalized Myasthenia Gravis)) OR (OMG)) OR (GMG) | 21582 |
| 3 | 1 OR 2 | 21582 |
| 4 | (SARS-CoV-2[MeSH Terms]) OR (COVID-19[MeSH Terms]) | 189101 |
| 5 | ((((((((COVID-19) OR (SARS-CoV-2)) OR (2019 Novel Coronavirus Disease)) OR (2019 Novel Coronavirus Infection)) OR (2019-nCoV)) OR (Coronavirus Disease 2019)) OR (Severe Acute Respiratory Syndrome Coronavirus 2)) OR (SARS Coronavirus 2) | 300071 |
| 6 | 4 OR 5 | 300071 |
| 7 | ((Vaccines[MeSH Terms]) OR (Vaccination[MeSH Terms])) OR (COVID-19 Vaccines[MeSH Terms]) | 296265 |
| 8 | (((Vaccines) OR (Vaccination)) OR (COVID-19 Vaccines)) OR (Active Immunization) | 475927 |
| 9 | 7 OR 8 | 475927 |
| 10 | 6 AND 7 | 44542 |
| 11 | 3 AND 10 | 56 |

Search strategy for Cochrane Library

| No. | Search (untagged = all searchable fields which includes titles, abstracts, and index terms) | Records Retrieved |
| --- | --- | --- |
| 1# | MeSH descriptor: [Myasthenia Gravis] explode all trees | 244 |
| 2# | (Myasthenia Gravis) OR (MG) OR (Ocular Myasthenia Gravis) OR (Generalized Myasthenia Gravis) OR (OMG) | 352949 |
| 3# | (GMG) | 135 |
| 4# | #1 OR #2 OR #3 | 352973 |
| 5# | MeSH descriptor: [SARS-CoV-2] explode all trees | 1120 |
| 6# | MeSH descriptor: [COVID-19] explode all trees | 2305 |
| 7# | (COVID-19) OR (SARS-CoV-2) OR (Novel Coronavirus) OR (Coronavirus Disease 2019) OR (Severe Acute Respiratory Syndrome Coronavirus 2) | 12896 |
| #8 | (SARS Coronavirus 2) | 3096 |
| #9 | #5 OR #6 OR #7 OR #8 | 12785 |
| #10 | MeSH descriptor: [Vaccines] explode all trees | 14163 |
| #11 | MeSH descriptor: [Vaccination] explode all trees | 2894 |
| #12 | MeSH descriptor: [COVID-19 Vaccines] explode all trees | 203 |
| #13 | (Vaccines) OR (Vaccination) OR (COVID-19 Vaccines) OR (Active Immunization) | 43548 |
| #14 | #10 OR #11 OR #12 OR #13 | 25262 |
| #15 | #4 AND #9 | 2512 |
| #16 | #14 AND #15 | 143 |

Search strategy for Web of Science

| No. | TS = title, abstract, author keywords, and Keywords Plus | Records Retrieved |
| --- | --- | --- |
| #1 | (((((TS=(Myasthenia Gravis)) OR TS=(MG)) OR TS=(Ocular Myasthenia Gravis)) OR TS=(Generalized Myasthenia Gravis)) OR TS=(OMG)) OR TS=(GMG) | 2321785 |
| #2 | ((((((TS=(SARS-CoV-2)) OR TS=(COVID-19)) OR TS=(2019 Novel Coronavirus)) OR TS=(2019-nCoV)) OR TS=(Coronavirus Disease 2019)) OR TS=(Severe Acute Respiratory Syndrome Coronavirus 2)) OR TS=(SARS Coronavirus 2) | 377140 |
| #3 | (((TS=(Vaccines)) OR TS=(Vaccination)) OR TS=(COVID-19 Vaccines)) OR TS=(Active Immunization) | 661237 |
| #4 | #1 AND #2 AND #3 | 455 |
